# Intrinsic Spectral Resolution Limitations of QEPAS Sensors for Fast and Broad Wavelength Tuning

**DOI:** 10.3390/s20174725

**Published:** 2020-08-21

**Authors:** Jesper B. Christensen, Lasse Høgstedt, Søren M. M. Friis, Jui-Yu Lai, Ming-Hsien Chou, David Balslev-Harder, Jan C. Petersen, Mikael Lassen

**Affiliations:** 1 Danish Fundamental Metrology, Kogle Allé 5, 2970 Hørsholm, Denmark; jbc@dfm.dk (J.B.C.); dbh@dfm.dk (D.B.-H.); jcp@dfm.dk (J.C.P.); 2 NLIR Aps, Hirsemarken 1, 3520 Farum, Denmark; lh@nlir.com (L.H.); sf@nlir.com (S.M.M.F.); 3 HC Photonics, 4F, No. 2, Technology Rd. V, Hsinchu City 300, Taiwan; jyl@hcphotonics.com (J.-Y.L.); mhc@hcphotonics.com (M.-H.C.)

**Keywords:** quartz-enhanced photoacoustic spectroscopy, quartz tuning fork, environmental sensor, optical parametric oscillator, molecular spectroscopy

## Abstract

Quartz-enhanced photoacoustic sensing is a promising method for low-concentration trace-gas monitoring due to the resonant signal enhancement provided by a high-Q quartz tuning fork. However, quartz-enhanced photoacoustic spectroscopy (QEPAS) is associated with a relatively slow acoustic decay, which results in a reduced spectral resolution and signal-to-noise ratio as the wavelength tuning rate is increased. In this work, we investigate the influence of wavelength scan rate on the spectral resolution and signal-to-noise ratio of QEPAS sensors. We demonstrate the acquisition of photoacoustic spectra from 3.1 μm to 3.6 μm using a tunable mid-infrared optical parametric oscillator. The spectra are attained using wavelength scan rates differing by more than two orders of magnitude (from 0.3 nm s${}^{-1}$ to 96 nm s${}^{-1}$). With this variation in scan rate, the spectral resolution is found to change from 2.5 cm${}^{-1}$ to 9 cm${}^{-1}$. The investigated gas samples are methane (in nitrogen) and a gas mixture consisting of methane, water, and ethanol. For the gas mixture, the reduced spectral resolution at fast scan rates significantly complicates the quantification of constituent gas concentrations.

## 1. Introduction

Detection and monitoring of hazardous chemicals and greenhouse gases is an increasingly important task [1,2]. Photoacoustic spectroscopy (PAS), in one of its many variations [3,4,5,6,7,8,9,10,11,12,13,14,15], is highly suitable for this task due to its high sensitivity. One of the PAS variants, QEPAS, was introduced in 2002 [16], and is today an established spectroscopic technique [17]. In QEPAS, amplitude- or wavelength modulated laser light generates an acoustic wave via excitation, and a following relaxation of a gas sample [18,19,20]. The acoustic wave is detected using a quartz tuning fork (QTF) with a high quality factor ($Q>10^{3}$) and an eigenfrequency matching the laser modulation frequency [16,21]. The QTF is usually complemented by longitudinal micro resonators (mR) providing acoustic guidance and further signal enhancement. The detected photoacoustic (PA) signal scales linearly with the quality factor, enabling this type of sensor to achieve extremely high sensitivities. Gas concentrations of parts per billion (ppb) and parts per trillion (ppt) have been detected with QEPAS sensors for many different types of gasses [11,12,17,22,23,24,25,26,27,28]. Moreover, the QEPAS sensor benefits from being highly immune to environmental noise sources. This stems in part from the small resonance bandwidth (a few hertz) of the QTF and in part from the construction requiring the QTF prongs to oscillate out of phase by $180^{\circ }$ to be piezo-electrically active [17,29]. External acoustic noise within the bandwidth of the QTF has a longer wavelength than the prong separation and will cause the prongs to oscillate in phase, resulting in no piezo-electric response.

Despite the large potential of QEPAS sensors, their reliability in real-life applications have barely been addressed in the literature [14,30,31,32]. Most experimental demonstrations have been conducted in a laboratory environment using a single gas in an N${}_{2}$ matrix. Applying QEPAS sensors to complex gas mixtures, necessitates spectroscopic analysis over large spectral ranges and fast tuning rates, in order to quantify individual gas concentrations in real time. However, at fast spectral tuning rates, QEPAS sensors have an intrinsic limitation in the spectral resolution (instrument profile). The reason behind this is intrinsically connected to its main advantage, namely the large QTF quality factor, which results in mechanical relaxation times of hundreds of milliseconds [33]. This leads to a degraded spectral resolution of the QEPAS sensor, when performing measurements at fast spectral tuning rates. Consequently, when performing measurements on a complex gas mixture, where cross-interference effects are present, it becomes difficult, or almost impossible, to identify individual gases and quantify their concentrations [34].

In this work, we aim to experimentally investigate how the sensitivity and resolution of broadband QEPAS sensors depend on the employed spectral scan rate. To this end, we demonstrate QEPAS measurements on a simple gas of (100±3) ppm methane in nitrogen and a “complex” gas mixture with (2500±150) ppm water vapor, (87±5) ppm methane, and (50±15) ppm ethanol in nitrogen. The measurements are all conducted in the mid-infrared (MIR) region from 3.1 μm to 3.6 μm enabled by a wavelength-tunable pulsed optical parametric oscillator (OPO). The PA spectra are acquired at varying scan rates from 0.3 nm s${}^{-1}$ to 96 nm s${}^{-1}$ and it is demonstrated that the spectral resolution and single-shot sensitivity of the QEPAS sensor depend critically on both the employed scan rate and the mechanical relaxation time of the QTF. In our case, when sweeping the wavelength at rates below 16 nm s${}^{-1}$ we find that our QEPAS spectrometer has a spectral resolution of 2.5 cm${}^{-1}$ limited by the excitation light from the MIR-OPO. By increasing the wavelength sweep rate to 96 nm s${}^{-1}$, the QEPAS spectral resolution decreases to 9 cm${}^{-1}$ as a result of the slow QTF relaxation.

## 2. Experimental Setup

**Figure 1:**
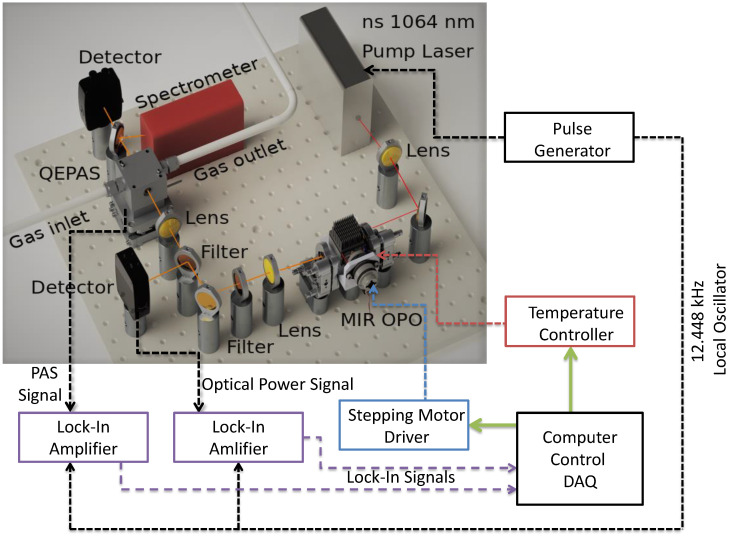
Schematics of the QEPAS sensor system with the MIR OPO and QEPAS cell (Thorlabs, ADM01) as the main elements.

The block diagram of the experimental setup is shown in Figure 1. The setup includes a home-built nanosecond MIR-OPO, tunable between 2.8 and 4.4 μm, a QEPAS cell, thermal detectors for monitoring the optical power, a signal generator for controlling the MIR-OPO repetition rate, a stepping motor driver, an optical spectrum analyser, pressure and temperature controllers for the QEPAS sensor and OPO, and lock-in amplifiers connected to a fast 12 bit oscilloscope for data acquisition. Below we describe the MIR-OPO and QEPAS cell in more detail.

### 2.1. The MIR OPO

**Figure 2:**
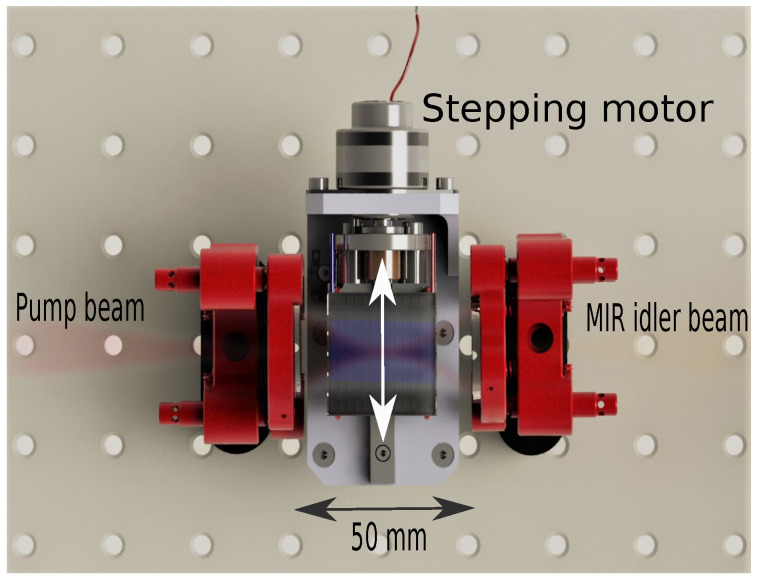
The MIR OPO is optimized for operation in the spectral region between 2.9 μm and 3.6 μm. The linear cavity consists of spherical mirrors each with a radius of curvature of 100 mm separated by 50 mm. The pulsed MIR source is characterized by a repetition rate of 12.448 kHz, a pulse duration of ∼40 ns, and a potential average output power in excess of 400 mW. A computer is controlling the stepping motor and temperature of the nonlinear crystal: a 30×20×1 mm${}^{3}$ periodically poled lithium niobate bulk crystal with a chirped fan-out structure of poling periods ranging from 27.4 μm to 31.75 μm.

**Figure 3:**
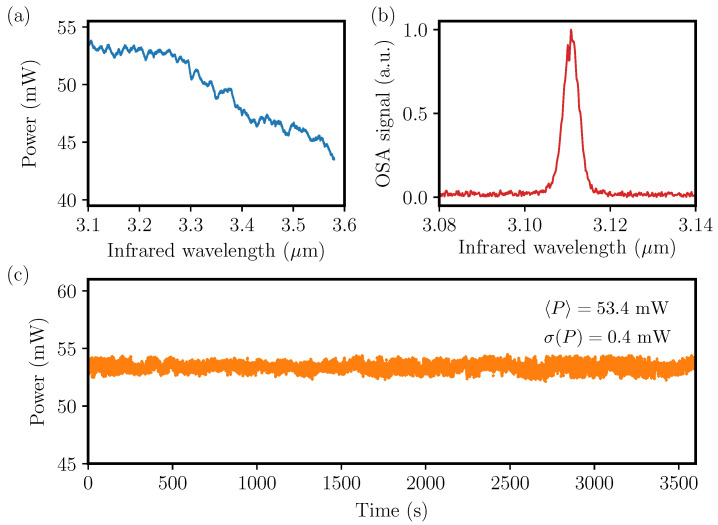
MIR-OPO performance characterization. (**a**) Average output power curve versus wavelength. (**b**) Example pulse spectrum measured at 3.11 μm. (**c**) Power-stability measurement at a fixed wavelength of 3.11 μm over a duration of 1 h.

For highly sensitive and selective PAS measurements it is desirable to have a MIR light source with high power, fast wavelength sweeping and large wavelength tunability covering the 2.8 μm to 4.4 μm region, where most trace gasses have strong vibrational transitions. For this a nanosecond pulsed MIR-OPO is a good candidate, since it provides good molecular selectivity, due to large wavelength tunability, relative narrow linewidth, and high optical output powers [34,35]. The MIR-OPO used is based on a 30 × 20 × 1 mm${}^{3}$ periodically poled lithium niobate (PPLN) nonlinear crystal with a fanned-out structure of poling period ranging from 27.4 μm to 31.75 μm (HC Photonics). The resulting wavelength tuning ranges for the NIR signal and MIR idler are 1.4 μm to 1.7 μm and 2.8 μm to 4.4 μm, respectively. The PPLN is placed inside a 50 mm long single-resonant cavity consisting of two identical spherical mirrors with radius of curvature of 100 mm. The mirrors have a high reflection coating for the signal wavelengths and an anti-reflection coating for the pump and idler wavelengths. Thus, the cavity is single-resonant for the generated near-infrared (NIR) signal beam, and the relatively simple design is shown in Figure 2. The MIR-OPO is pumped with an actively Q-switched 1064-nm diode-pumped nanosecond laser (Bright Solutions), allowing the repetition to be continuously changed from single-shot to 100 kHz. At a repetition rate of 12.448 kHz, the pulse duration of the MIR idler is approximately 40 ns and can reach an average power in excess of 400 mW at maximum pump power. The continuous tuning of the repetition rate enables synchronization of the repetition rate of the MIR-OPO with the acoustic resonance of the QTF, thus being similar to an amplitude modulated continuous-wave light QEPAS setup. The QEPAS signal is processed with a lock-in amplifier using 1-f demodulation. The tunability of the OPO is achieved by perpendicular translation of the fanned-out PPLN crystal using a standard bipolar stepper motor (G4 25000, Haydon Kerk Motion Solutions, Waterbury, CT, USA), while the temperature of the crystal is kept near 30 ${}^{\circ }$C. In this way wavelength sweeping rates in excess of 100 nm s${}^{-1}$ can be archived. Figure 3 shows the performance data of the MIR OPO. Figure 3a shows the mean MIR optical power as function of wavelength. The optical power is varying slightly over the tuning range. This needs to be taken into account when performing spectroscopic QEPAS measurements through normalization of the PA signal with the optical power. Figure 3b shows the MIR pulse measured with Fourier transform optical spectrum analyzer with a resolution of 0.25 cm${}^{-1}$. The full-width at half maximum is approximately 4 cm${}^{-1}$, and varies only slightly with wavelength. Figure 3c shows the power stability of the MIR-OPO when operating at 3.11 μm over 1 h of measurement. The mean optical power used in the experiments below was approximately 53 mW (at λ=3.11μm), at which we estimate a standard deviation of 0.4 mW (less than 1%).

### 2.2. The QEPAS Cell

The PA technique is based on the detection of sound waves that are generated due to molecular absorption of modulated radiation. The voltage magnitude of the measured PA signal is given by [19]:

(1) $$S_{\mathrm{PA}}=PF\alpha ,$$

where *P* is the average power of the incident radiation, α is the absorption coefficient, which depends on the total number of molecules per cm${}^{3}$ and the absorption cross section, and *F* is a cell-specific constant, which depends on the size and geometry of the cell, the modulation frequency, the efficiency of the transducer, and, in the QEPAS variation, the quality factor *Q* of the acoustic resonance. To evaluate the performance of the QEPAS sensor the sensitivity can be quantified by a figure of merit, called the normalized noise equivalent absorption (NNEA). The NNEA represents the minimum detectable absorption in units of inverse length, normalized to the laser power and the electrical measurement bandwidth. It is defined as follows [24]:

(2) $$\mathrm{NNEA}=\frac{P\times \alpha }{\mathrm{SNR}\times \sqrt{\Delta f}}\;,$$

where *P* is the average optical power in watts; α is the target gas absorption in ${\mathrm{cm}}^{-1}$; Δf is the detection bandwidth in Hz; and SNR is the signal-to-noise ratio, taken as the ratio of the signal value with the root-mean-square noise within the same bandwidth.

**Figure 4:**
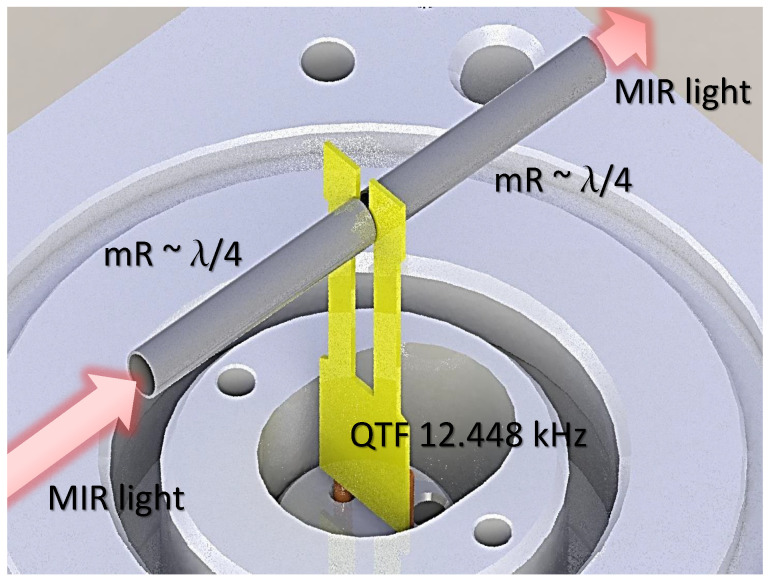
Schematics of the QEPAS cell. The QEPAS cell used for these tests is commercially available (Thorlabs, ADM01). Two micro-resonator (mR) tubes are aligned perpendicular to the QTF plane to probe the acoustic vibration excited in the gas. The length of the mR tubes is 7 mm and the resonant frequency of the QTF is 12.448 kHz. With a gas sample volume of 7 cm${}^{3}$. The windows on the cell are made of uncoated BaF${}_{2}$ with a transmission of 95%.

The used PA cell (Thorlabs, ADM01), is designed for on-axis QEPAS and uses two mR tubes on either side of the QTF as an acoustic resonator as shown in Figure 4. Each tube has an inner diameter of 1.6 mm and a length of $\lambda_{s}/4$ with a small gap between them for the QTF, where $\lambda_{s}$ is the wavelength of a sound wave in air at the resonance frequency of the tuning fork ($f_{0}=12.448$ kHz). The acoustic mRs increase the effective interaction length between the generated sound and the QTF, allowing the QTF to have higher sensitivity to the near-field photoacoustic wave [22]. The gas sample volume is ∼7 cm${}^{3}$. The optical transmission is approximately 90% and the optical power is monitored by detectors placed before and after the QEPAS cell. The piezo-transducer electrical signal from the QTF is amplified by an integrated high-gain transimpendance pre-amplifier, before being processed with a lock-in amplifier, and finally digitized with a fast 12-bit oscilloscope.

## 3. Results

### 3.1. Slow Wavelength Sweep

**Figure 5:**
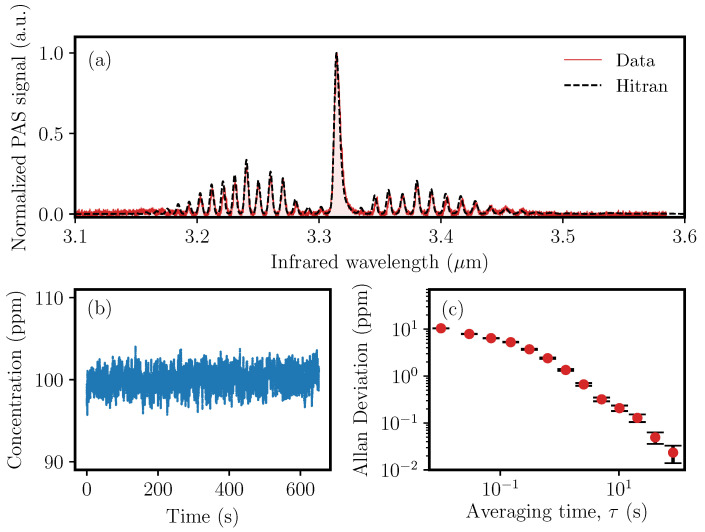
(**a**) Photoacoustic spectroscopic data for 100 ppmV CH${}_{4}$ in an N${}_{2}$-matrix at a pressure of 800 mbar (red) referenced against Hitran data for methane absorption using a Gaussian instrumental profile of 2.5 cm${}^{-1}$ (black). The stepper-motor scan time is approximately half an hour corresponding to a wavelength scanning speed of 0.3 nm s${}^{-1}$. (**b**) Time data when the OPO is locked to the the Q-branch absorption peak at 3.32 μm. The standard deviation is 1 ppm. (**c**) The corresponding Allan deviation shows that the detection limit for methane is 23 ppbV (nmol/mol) for 82 s of integration time.

The first QEPAS experiments were performed by excitation of molecular ro-vibrational transitions of 100 ppmV of methane (CH${}_{4}$) in nitrogen (N${}_{2}$) in the 3.1–3.6 μm wavelength region. Figure 5a shows the acquired photoacoustic spectrum, which clearly allows identification of the R-, Q- and P-branches of CH${}_{4}$. The data were processed with a lock-in amplifier measuring the 1st harmonic of the generated PAS signal with a time constant of 10 ms. The wavelength of the OPO is changed with a speed of approximately 0.3 nm s${}^{-1}$, corresponding to a single-sweep duration of around 28 min, where the PAS signal is generated by pure amplitude modulation, due to the ns pulses with a repetition rate matching the QTF resonance. The experimental data is compared to methane data from the Hitran database applying a Gaussian instrumental profile of 2.5 cm${}^{-1}$. Comparison with the Hitran database CH${}_{4}$ absorption spectra requires conversion of the PA time trace to wavelength (or wavenumber). This time-to-wavelength conversion is accomplished using numerically calculated phase-matching curves for PPLN [36] while assuming a constant stepper-motor speed and a linear chirp of the PPLN poling period. The remarkable agreement between the attained photoacoustic spectrum and Hitran data seen in Figure 5 demonstrates the validity of this approach.

Characterization of long-term drifts and signal averaging limits is a very important measure for all types of sensors. There exists an optimum integration time at which the detection limit reaches a minimum value. At longer averaging time, drift effects emerge and the sensor performance deteriorates. The optimum integration time and detection sensitivity are determined using an Allan deviation analysis. The MIR-OPO is tuned to the Q-branch absorption peak at 3.32 μm. Figure 5b shows the time data with a mean methane concentration of 100 ppmV and with a standard deviation of 1 ppm. Figure 5c shows the corresponding Allan deviation. The Allan deviation analysis shows that the detection limit for methane is approximately 23 ppbV (nmol/mol) for 82 s of integration time, corresponding to a normalized noise equivalent absorption (NNEA) coefficient of $\left(1.5\pm 0.2\right)\times 10^{-8}$ W cm${}^{-1}$ Hz${}^{-1/2}$.

### 3.2. Fast Wavelength Sweep

**Figure 6:**
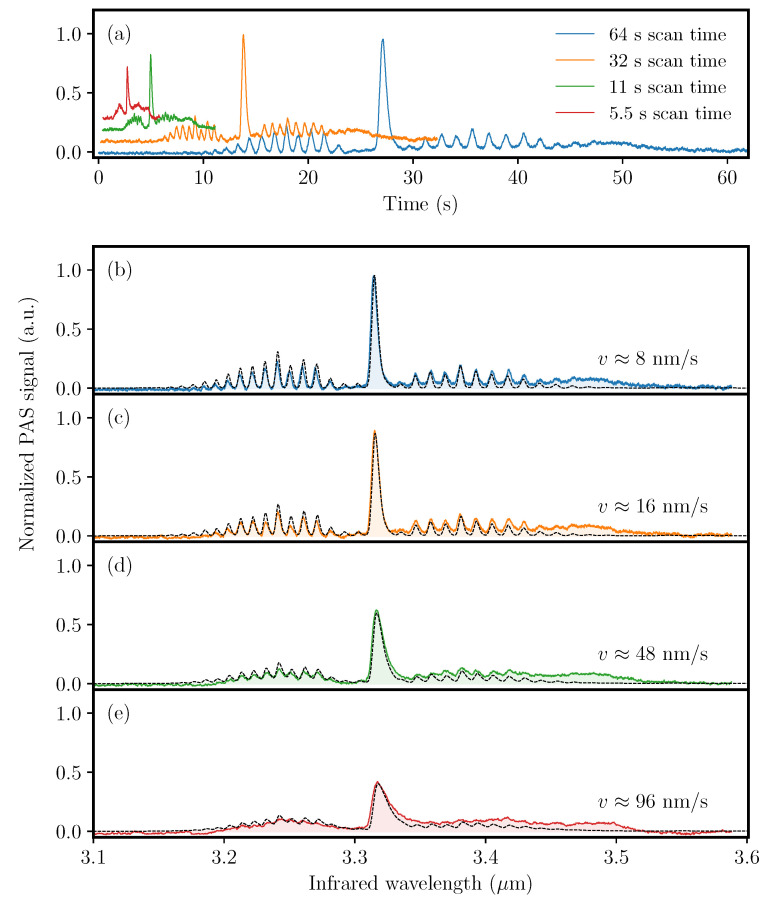
Acquired photoacoustic signal for 100 ppmV CH${}_{4}$ in an N${}_{2}$-matrix. (**a**) Photoacoustic signal versus time showing the relative time scales of the four stepper-motor speeds of 8 nm s${}^{-1}$, 16 nm s${}^{-1}$, 48 nm s${}^{-1}$, and 96 nm s${}^{-1}$. (**b**–**e**) Same data (color coded) as in (**a**), but plotted with respect to wavelength rather than time. For reference purposes, the black dashed lines represent Hitran absorption data for CH${}_{4}$ using appropriate instrumental profiles as explained in the text.

The QEPAS sensor performance was also investigated at faster wavelength scan rates using the same gas sample and wavelength range as above. Four resulting photoacoustic time traces are shown in Figure 6a with scan times of the 500-nm spectral bandwidth ranging from ∼1 min for the slowest scan and down to 5 s for the fastest. Figure 6b–e show the resulting photoacoustic spectra in reference to methane absorption data from the Hitran database. The two slowest scans, Figure 6b,c, result in photoacoustic spectra that adequately resolves the structure of the P- and R-branches. However, as the scanning rate is increased the spectral features become smeared out and gradually more difficult to resolve. Moreover, the spectra clearly illustrates the QTF relaxation effect upon excitation of the absorption lines in the Q-branch. The induced asymmetry reveals that the wavelength scan, as all others presented, is performed by scanning from 3.1 μm to 3.6 μm.

The Hitran data shown in Figure 6 is obtained through two successive convolutions of the line intensity spectrum S(ν) with instrumental response functions, i.e., $S\left(\nu \right)*h_{1}\left(\nu \right)*h_{2}\left(\nu ,v_{\nu }\right)$. The instrument functions are given as $h_{1}\left(\nu \right)\propto \exp\left[-\nu^{2}/\left(\Delta \nu^{2}\right)\right]$ and $h_{2}\left(\nu ,v_{\nu }\right)\propto \exp\left[-\nu /\left(v_{\nu }\tau_{\mathrm{QTF}}\right)\right]$ and describe, respectively, the effect of the excitation pulse bandwidth and the delayed response of the QTF. The QTF response depends on the (variable) scan rate $v_{\nu }$ and the decay time of the mechanical oscillation $\tau_{\mathrm{QTF}}=Q/\left(\pi f_{0}\right)$. At low scan rates, the impact of the delayed QTF response is negligible, and we find that $\Delta \nu =2.5\;{\mathrm{cm}}^{-1}$ provides a good fit to all acquired data. Increasing the scan rate $v_{\nu }$ allows determination of $\tau_{\mathrm{QTF}}$ as the QTF relaxation becomes apparent and reduces the spectral resolution. We find a best estimate for $\tau_{\mathrm{QTF}}$ of 75 ms based both on the relative PA peak values, and the signal decay times. The spectral resolution is dependent on the scan rate $v_{\nu }$; however, it only becomes significant at rates fullfilling $v_{\nu }\tau_{\mathrm{QTF}}≳\Delta \nu$. This approximate inequality provides a guide to the maximum scan rate that can be used without significantly reducing the spectroscopic resolution. In the present case, it is estimated that the spectral resolution decreases for scan rates exceeding $v_{\nu }\approx 33\;{\mathrm{cm}}^{-1}\;s^{-1}$ ($v_{\lambda }∼36\;\mathrm{nm}\;s^{-1}$). At the fastest scan rate used in this work, we find that the spectral resolution has reduced to 9 cm${}^{-1}$.

Apart from decreasing the spectral resolution of the PAS measurement, increasing the scanning rate has the additional effect of decreasing the peak value, and hence, the SNR [33]. This is evident from Figure 6b–e as the relative Q-branch signal decreases as the scanning speed is increased. This is another consequence of the damped resonant system, which requires a certain number of resonant oscillations in order to reach saturation at a given driving force. Explicitly we find that the SNR, given as the peak-to-noise of the Q-branch peak, for the slow (resolution of 2.5 cm${}^{-1}$) and fast (resolution of 9 cm${}^{-1}$) measurements are 197 and 87 respectively. The single-shot methane sensitivities in these two cases are therefore 0.5 ppmV and 1.1 ppmV, respectively. These values are, as expected, significantly lower than the long-term detection limit of 0.023 ppmV found using the Allan-deviation analysis above.

### 3.3. Investigation of a Gas Mixture: CH${}_{4}$, C${}_{2}$H${}_{6}$O, and H${}_{2}$O in N${}_{2}$

The industrial adoption of QEPAS sensors, among other things, relies on their ability to analyse gaseous mixtures, which contain species that cause spectroscopic interference [37,38,39,40]. Moreover, the presence of certain species enhances the strength of the photoacoustic signal from other species by influencing the molecular relaxation process [31,41,42]. As an example, a variation in water concentration causes a variation in the methane PA signal, which is not related to a change in the CH${}_{4}$ concentration. Thus, in order to guarantee reliable CH${}_{4}$ concentration measurements, the water-vapor concentration must be monitored simultaneously [43].

**Figure 7:**
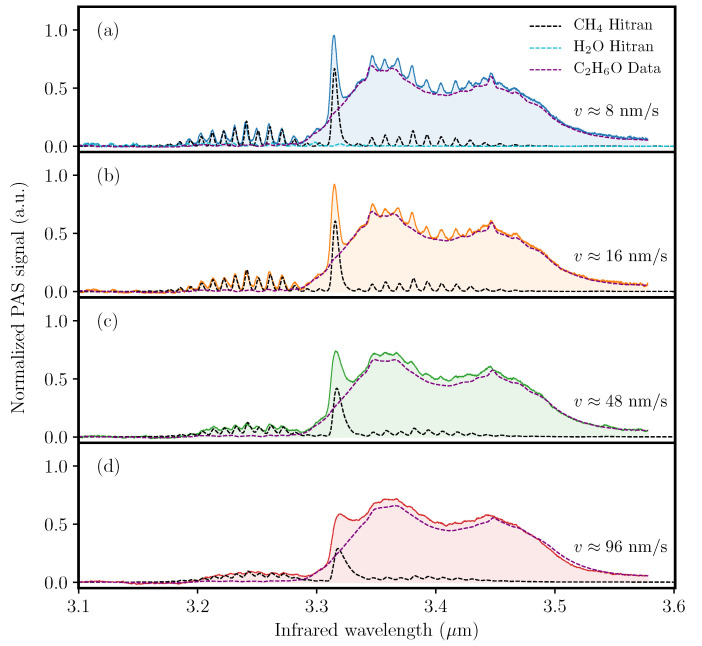
Acquired QEPAS spectra of approximately (87 ± 5) ppm of methane in a gaseous mixture with evaporated ethanol and water. The spectra were obtained using wavelength scan rates of (**a**) 8 nm s${}^{-1}$, (**b**) 16 nm s${}^{-1}$, (**c**) 48 nm s${}^{-1}$, and (**d**) 96 nm s${}^{-1}$. The black dashed curves represent methane and the cyan dashed curve represent the water absorption data from the Hitran database with an appropriate instrumental profile (see text). The purple dashed curves represent measured photoacoustic spectra for ethanol.

Figure 7 exemplifies a real-life scenario, in which the investigated gas is a mixture containing water, methane and ethanol (C${}_{2}$H${}_{6}$O). Prior to recording the illustrated spectra, we measured the photoacoustic spectrum of ethanol vapor. For each scan rate *v*, the ethanol data is convoluted with the response function $h_{2}\left(\nu ,v_{\nu }\right)$ and included in the corresponding spectrum (gray) for reference purposes. Moreover, the presence of water vapor is indicated by the small absorption features in the wavelength range from 3.1 μm to 3.15 μm. Hitran absorption data of H${}_{2}$O is also included, and, besides the absorption features in the low wavelength region, is seen to cause cross-interference with the R-branch of methane.

From Figure 7 it can be seen that the PA signal of methane’s Q-branch quickly decreases as the wavelength scan rate increases. On the other hand, the PA signal stemming from the broadband ethanol absorption remains largely unaffected. For the spectroscopic measurement of water vapor the effect is even more dramatic as the signal completely disappears in noise at wavelength scan rates of 48 nm s${}^{-1}$ and 96 nm s${}^{-1}$. These effects, on top of the spectral smearing, complicates the spectroscopic identification and quantification of constituents in a gaseous mixture. However, by using the obtained data for (87±5) ppm of methane to estimate the concentration of the unknown amounts of water and ethanol we find that their respective concentrations are (2500±150) ppm and (50±15) ppm.

## 4. Discussion

We have tested a QEPAS cell pumped resonantly by a nanosecond pulsed MIR-OPO for fast spectral data acquisition. The spectral branches of methane (CH${}_{4}$) are resolved, easily identified, and compared with the HITRAN database. The single-shot spectroscopic methane measurements have a detection sensitivity of approximately 0.5 ppmV. However, by using a longer integration time, the sensitivity can be improved to 23 ppbV at 82 s of averaging, corresponding to a normalized noise equivalent absorption coefficient of 1.5 × 10${}^{-8}$ W cm${}^{-1}$ Hz${}^{-1/2}$.

Our developed motorized MIR-OPO QEPAS system allows broad wavelength scans from 2.9 μm to 4.4 μm with a tunable wavelength-scanning rates up to 96 nm s${}^{-1}$. In the present investigation, we limited the MIR-OPO scanning range to the absorption of methane in the range 3.1 μm to 3.6 μm. Using the system, we estimated a resolution bandwidth of approximately $2.5\;{\mathrm{cm}}^{-1}$ for wavelength-scanning rates below approximately 16 nm s${}^{-1}$, limited by the spectral width of the optical pulses used for excitation. For increased wavelength scan rates we observed a gradual decrease in the spectral resolution. The asymmetric nature of the acquired spectra at fast scan rates clearly indicates that the decreased spectral resolution stems from the slow QTF damping. From the bandwidth of the excitation light we estimate that the spectral resolution is penalized for scanning rates exceeding ∼30 nm s${}^{-1}$, in fine agreement with the observed spectra. In this regard, it is noted that the wavelength scan rate at which the spectroscopic resolution begins to be affected, depends on the bandwidth of the excitation light source. For example, with the investigated QEPAS module (in its current state), the use of a quantum-cascade laser with a narrow bandwidth of $0.25\;{\mathrm{cm}}^{-1}$, would result in PA spectra with decreasing resolution as the scan rate exceeds 2 nm s${}^{-1}$.

The QEPAS sensor was also used to investigate a gaseous mixture consisting of water vapour, methane and ethanol vapor. These three gases exhibit a significant spectral overlap, and therefore the spectra are characterized by a strong degree of cross-interference. To test the ability of our QEPAS sensor to spectroscopically analyse the sample gas, we acquired four spectra using widely different wavelength scan rates of 8 nm s${}^{-1}$, 16 nm s${}^{-1}$, 48 nm s${}^{-1}$, and 96 nm s${}^{-1}$. Besides significantly reducing the spectral resolution of the acquired spectra, increasing the wavelength scan rate above 30 nm s${}^{-1}$ was also observed to reduce the single-shot measurement sensitivity. Both of these effects act to reduce the ability to quantify individual gas concentrations, and it is, therefore, highly desirable to not operate the investigated QEPAS sensor with scanning rates exceeding 30 nm s${}^{-1}$. With a scan rate of 30 nm s${}^{-1}$, the acquisition of PA spectra covering 500 nm could be done in less than 20 s. While this is too slow to be applied for gas emission burst detection, it would suffice for monitoring slow changes originating e.g., due to a commencing gas leak.

The advent of QEPAS sensors for practical environmental measurements requires the capability to differentiate between different gaseous species. This differentiation may only be performed through spectroscopic identification, which relies on the ability to cover a broad spectral range in a reasonable amount of time. However, as demonstrated, fast wavelength scan rates smear out the, otherwise distinctive, spectral features of the absorption lines. While the resulting increased spectral overlaps may potentially be dealt with using advanced data processing algorithms [44], it renders QEPAS sensors less suitable for providing broadband continuous spectra compared to other PAS methods. Instead, a viable approach could be to acquire PA signals at distinct, carefully considered, discrete wavelength points [45]. Another approach might be to design a system with a lower mechanical quality factor to allow for an increase in the applicable wavelength scan rate. This solution could especially be compelling in scenarios that requires the measurement of low- and moderate, rather than ultra-low, trace-gas concentrations.

## Abbreviations

The following abbreviations are used in this manuscript:

MIR	Mid-infrared
NNEA	Normalized noise equivalent absorption
OPO	Optical parametric oscillator
PA	Photoacoustic
PAS	Photoacoustic spectroscopy
QEPAS	Quartz-enhanced photoacoustic spectroscopy
QTF	Quartz tuning fork
